# Exploring moisture adsorption on cobalt-doped ZnFe_2_O_4_ for applications in atmospheric water harvesting†

**DOI:** 10.1039/d3ra08152d

**Published:** 2024-02-19

**Authors:** Muhammad Ehtisham, Ahmad K. Badawi, Asad Muhammad Khan, Rafaqat Ali Khan, Bushra Ismail

**Affiliations:** a Department of Chemistry, COMSATS University Islamabad, Abbottabad Campus 22060 Pakistan muhammadehtasham10@gmail.com amkhan@cuiatd.edu.pk rafaqatali@cuiatd.edu.pk bushraismail@cuiatd.edu.pk +92 992 383595 +92 992 383592; b Civil Engineering Department, El-Madina Higher Institute for Engineering and Technology Giza 12588 Egypt dr.ahmedkaram91@gmail.com

## Abstract

Sorption-based atmospheric water harvesting (SBAWH) is a highly promising approach for extracting water from the atmosphere thanks to its sustainability, exceptional energy efficiency, and affordability. In this work, ZnFe_2_O_4_ and Zn_0.4_Co_0.6_Fe_2_O_4_ were evaluated for moisture adsorption. The desired materials were synthesized by a surfactant-assisted sol–gel method. Synthesized samples were characterized using X-ray diffraction (XRD) analysis, scanning electron microscopy (SEM), energy dispersive X-ray (EDX) spectroscopy, Fourier transform infrared (FTIR) spectroscopy, vibrating sample magnetometry (VSM), and point of zero charge (PZC). Crystallinity and phase composition were evaluated by XRD analysis. Several parameters were determined using XRD analysis: lattice parameter, unit cell volume, crystallite size, and bulk density. The morphology of synthesized materials was assessed *via* SEM, and unveiled the acquisition of consistent, homogeneous, and uniform crystals. Elemental composition was determined through EDX spectroscopy. Water adsorption on the surface was evaluated by FTIR spectroscopy. The magnetic properties of synthesized ZnFe_2_O_4_ and cobalt-doped ZnFe_2_O_4_ ferrites were investigated using VSM. The negative charge on the Zn_0.4_Co_0.6_Fe_2_O_4_ surface was explored using PZC. Adsorption studies on synthesized materials were conducted with the help of an atmospheric water harvesting (AWH) plant created by our team. Moisture adsorption isotherms of synthesized materials were determined using a gravimetric method under varying temperature and relative humidity (45–95%) conditions. The moisture content (*M*_c_) of Zn_0.4_Co_0.6_Fe_2_O_4_ and ZnFe_2_O_4_ was 597 mg g^−1^ and 104 mg g^−1^, respectively. Key thermodynamic properties, including isosteric heat of adsorption (*Q*_st_), change in Gibbs free energy (Δ*G*), and change in sorption entropy (Δ*S*), were evaluated. *Q*_st_ was negative, which confirmed the sorption of water vapors on the material surface. Δ*G* and Δ*S* indicated that water-vapor adsorption was spontaneous and exothermic. A second-order kinetics study was carried out on synthesized materials, demonstrating their chemisorption behavior. The latter was due to the oxygen defects created by replacement of Co^2+^ and Fe^3+^ at tetrahedral and octahedral sites. Water vapors in the atmosphere became attached to the surface and deprotonation occurred, and the hydroxyl ions were formed. Water vapor attached to these hydroxyl ions. A second-order kinetics study was carried out to confirm the chemisorption behavior of synthesized materials.

## Abstract 2

Sorption-based atmospheric water harvesting (SBAWH) is a highly promising approach for extracting water from the atmosphere thanks to its sustainability, exceptional energy efficiency, and affordability.

## Graphical abstract

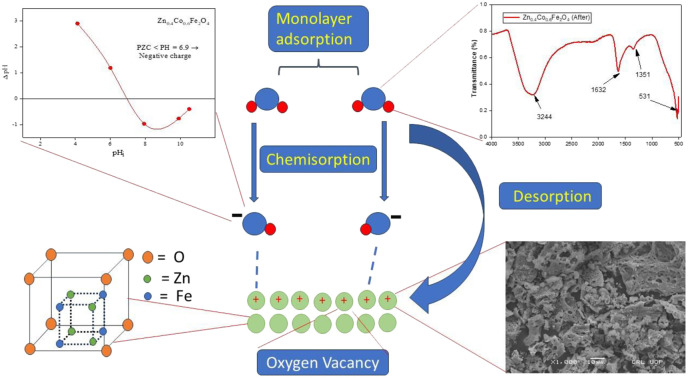

## 1. Introduction

The most precious source and a necessity for human survival is water. Worldwide water scarcity has reached an alarming situation. Many human lives worldwide are at risk due to an acute shortage of water. Several methods to obtain fresh water have been reported, the most prominent of which are desalination and wastewater treatment. However, they either require high energy to operate or have high costs. Only 2.5% of the total water worldwide is considered to be fresh water.^1^ Additionally, ∼13 000 km^3^ of water is present in the atmosphere in the form of vapor. According to a World Health Organization (WHO) survey, 2.5% of water is thought to be freshwater (comprising ice and snow (70%) and groundwater (30%)).^2^

According to another survey, the total water resources worldwide are 13 × 10^8^ km^3^. Only 35 million km^3^ (or 2.5%) of water is considered to be freshwater, the remaining part of water comprising oceans, rivers, seas, and lakes. A large amount of water (24 million km^3^ or 68.7%) comes from glaciers and ice caps in Antarctic and Arctic regions. The main sources for human consumption of freshwater are lakes and rivers, which contain ∼90 000 km^3^ (or 0.26%) of total water resources. The total amount of freshwater in the atmosphere is ∼13 000 km^3^ (or 0.04%) of total freshwater. Out of this 0.04% of atmospheric water, 69% comes from permafrost regions, 20.9% from lakes, 3.8% from soil moisture, 2.6% from bogs and marshes, and 0.49% from oceans.^3^

According to the WHO, only 71% of the worldwide population (or ∼5.3 billion people) have access to safe drinking water, whereas the remaining 2.2 billion people do not. Approximately 1.4 billion people can obtain water only from an improved water source located within 30 min of their location, and 785 million people do not have access to the most basic type of drinking water. Among the 206 million people who must collect water for >30 min, 435 million use unprotected springs and wells, and 144 million rely on untreated surface water deposited from lakes, ponds, and rivers.^4^

Water scarcity has also been increased due to contaminated or unsafe water. Contaminated water has a negative impact on human health, resulting in 14 000 daily deaths worldwide. The main cause of water contamination is the incomplete disposal of domestic and industrial waste onto surface water. This waste contains a large quantity of toxic substances, including harmful bacteria and heavy metals. The concentration of heavy metals such as lead (Pb), cadmium (Cd), and arsenic (As) above the safe limit has very adverse effects on human and aquatic life. For example, if the concentration of arsenic in groundwater is >50 μg L^−1^, then skin pigmentation, keratosis, and chronic cough can result. Several facial composite adsorbents^5^ have a high adsorption capacity of Cd(II),^6^ Ni(II),^7^ Ce(III),^8^ As(III),^9^ Sm(III),^10^ Cu(II),^11–13^ Cs(I),^14^ Eu(III)^15^ and methyl orange (MO) from contaminated water.^16^ Organic waste in groundwater leads to the growth of worms, viruses, and bacteria that are dangerous to human health.^17^ More than 20% of projected global needs will be met by the projected water shortage by 2030, which is close to 2000 billion m^3^.

There are various methods to obtain freshwater, such as wastewater treatment and desalination. Desalination has several advantages, such as high adsorption capacity. Each process requires high cost and high energy, which limits their application towards water harvesting. An effective method for water harvesting is sorbent-based atmospheric water harvesting (SBAWH). This approach offers an array of benefits, ranging from affordability and ease of synthesizing essential materials to reduced energy demands for water desorption and a minimal environmental impact. Remarkably, SBAWH is applicable in arid regions or areas with low relative humidity, making it a practical solution where other methods may falter.^18^

Numerous adsorbent materials are employed in SBAWH, including metal–organic frameworks (MOFs), hygroscopic salts, silica gel, and zeolites. These adsorbents exhibit impressive water-adsorption capacities, but they are not without limitations, which points to a significant research gap. For instance, MOFs are known for their high cost and complexity of their preparation, which hinders their practical application. On the other hand, hygroscopic salts are prone to deliquescence, which poses challenges to the consistency of their performance. The adsorption capacity of the zeolite AlPO4-LTA is 370 mg g^−1^ at 70% relative humidity (RH),^19^ which is less than that of the synthesized Zn_0.4_Co_0.6_Fe_2_O_4_ at 95% RH (present work). The water-vapor regeneration temperature of silica gel and zeolites is even higher (100–200 °C),^20^ making them an energy-intensive adsorbent material,^21^ while it is even less for Zn_0.4_Co_0.6_Fe_2_O_4_ (45 °C) (present work).

We aimed to explore whether, as alternatives to conventional adsorbent materials, ZnFe_2_O_4_ and cobalt-doped ZnFe_2_O_4_ were more cost-effective, stable, environmentally friendly, non-toxic, and required a lower regeneration temperature. The objective is to assess their potential in SBAWH considering their unique properties and cation redistribution within their structure, which generate positive and negative charges on their surfaces. These charges are believed to have a critical role in the adsorption of water vapors.

## 2. Experimental

### 2.1 Synthesis

The starting materials used during the synthesis of ZnFe_2_O_4_ and Zn_0.4_Co_0.6_Fe_2_O_4_ were zinc nitrate hexahydrate Zn(NO_3_)_2_·6H_2_O, iron nitrate nonahydrate Fe(NO_3_)_3_·9H_2_O, cobalt nitrate hexahydrate Co(NO_3_)_2_·6H_2_O, ethylenediamine tetra-acetic acid (EDTA), ethanol (CH_3_CH_2_OH), cetyltrimethylammonium bromide (CTAB), and ammonium hydroxide (NH_4_OH). All of these materials were of high purity and purchased from Daejung.

The surfactant-assisted sol–gel method was used to synthesize ZnFe_2_O_4_ and Zn_0.4_Co_0.6_Fe_2_O_4_. In this method, a 0.3 M (2.63 g) EDTA solution in 30 mL of ammonia water was prepared so that the molar ratio of cations and EDTA was equal.^22^ This EDTA solution was placed on a hot plate for continuous stirring. Next, a 0.1 M (2.97 g/100 mL) solution of zinc precursor and 0.2 M (8.08 g/100 mL) solution of the iron precursor was added dropwise to an EDTA solution. The temperature of the solution was maintained at 70 °C with constant stirring.^23^ Then, a 0.01 M (36 mg/10 mL) solution of CTAB was prepared by using ethanol as a solvent. Then, this 0.01 M solution of CTAB was added dropwise to a solution. After adding a surfactant, the temperature of the solution was maintained at 80 °C at constant stirring until a homogenous mixture was obtained. Then, the pH of the solution was adjusted to 9 by adding ammonia water dropwise.^24^ Next, the mixture was heated at 80 °C for 4 h until a viscous gel was formed. Then, this gel was placed overnight for aging. Next, the obtained viscous gel was dried at 200 °C.^23^ Finally, the dry gel was formed, and this dry gel was converted into powder using a mortar and pestle. The obtained ZnFe_2_O_4_ powder was washed using ethanol to remove excess surfactant.^25^ Then, the washed ZnFe_2_O_4_ powder was calcined at 800 °C for 8 h.^23^

To prepare 60% cobalt-doped ZnFe_2_O_4_ samples, a 0.3 M (2.63 g/30 mL) EDTA solution in 30 mL of ammonia was prepared and kept on a hot plate. Next, a 0.06 M (1.74 g/100 mL) solution of cobalt nitrate hexahydrate, 0.04 M (1.19 g/100 mL) solution of zinc nitrate hexahydrate, 0.2 M (8.08 g/100 mL) solution of iron nitrate nonahydrate were mixed dropwise in an EDTA solution. Then, the same procedure as mentioned above was followed.

### 2.2 Characterization

Phase composition and crystallinity were evaluated by XRD analysis. The lattice parameter (*a*), cell volume (*V*_cell_), crystallite size (*D*), and X-ray density (*ρ*_X-ray_) of desired samples were obtained using the following equations:

1 *a* = [*d*^2^(*h*^2^ + *k*^2^ + *l*^2^)]^1/2^

2 *V*_cell_ = *a*^3^

3
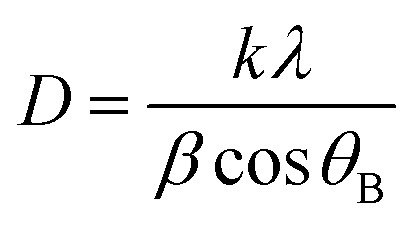
4
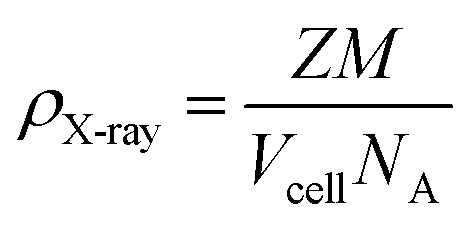
where “*d*” is the value of *d*-spacing between diffraction lines, “*hkl*” are the values of Miller indices, “*b*” is the diffraction broadening measured at full-width half maximum (FWHM), “*k*” is the wavelength of X-rays, “*θ*_B_” is the Bragg's angle, “*K*” is a constant (0.9 for a cubic system), “*Z*” is the number of molecules per formula unit (*Z* = 8 for spinel system), “*M*” is the molar mass, “*V*_cell_” is the cell volume, and “*N*_A_” is the Avogadro number. Surface morphology was obtained by scanning electron microscopy (SEM). The elemental composition was obtained using energy-dispersive X-ray spectroscopy.

### 2.3 Point of zero charge (PZC)

PZC was calculated using the salt-addition method. Briefly, 12.5 mg of the sample was mixed in 5 mL of a 0.1 M solution of KNO_3_ and the pH maintained at 2, 4, 6, 8, or 10. The initial pH (pH_i_) was adjusted by adding a 0.1 M solution of HCl and 0.1 M solution of NaOH dropwise. After maintaining the desired pH, the sample was placed on an orbital shaker for 24 h to determine the change in pH (ΔpH). Plotting a graph between the change in pH (ΔpH) *versus* initial pH (pH_i_) gave the pH_pzc_. The charge on the surface is negative if pH > pH_pzc_ and positive if pH < pH_pzc_.^26^

### 2.4 Adsorption studies

**Fig. 1:**
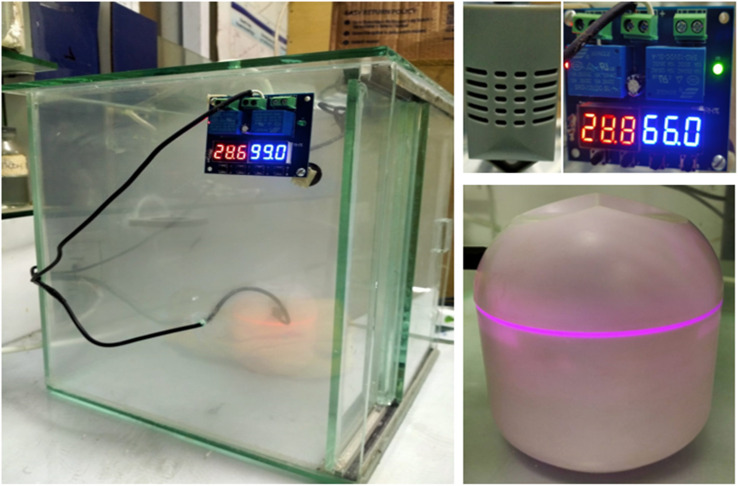
Atmospheric water-harvesting plant developed by our team.

The initial weight of as-prepared samples was measured. Samples were washed with deionized water to remove impurities from the surface, followed by filtration and drying in an oven at 80 °C until a constant weight was achieved. An experiment based on water-vapor adsorption was carried out using an atmospheric water-harvesting (AWH) plant designed by our team. This is a simple closed apparatus made up of glass boundary, and a humidifier is installed in it to control humidity. Furthermore, a small fan was installed to maintain humidity and temperature in a closed cabin. A small bulb was employed to increase the temperature within the isolated cabin (Fig. 1). The equilibrium moisture content (EMC) of ZnFe_2_O_4_ was determined at humidity levels from 45% to 95%. The total moisture content in the material was determined using the standard gravimetric method. Initially, synthesized samples were dried in an oven at 200 °C for 2 h to remove the initial water present in samples. Adsorption studies were done in an isolated cabin with controlled humidity ranging from 45% to 95%. Briefly, samples were placed in an isolated cabin in the AWH plant at a particular humidity. Weight change was observed every 20 min until no further adsorption occurred, which was considered the saturation point. The EMC of all samples was the maximum water adsorbed at saturation point. The EMC of as-synthesized samples at different humidity levels could also be achieved by the above-mentioned procedure. Each experiment was executed meticulously on two separate occasions to ensure the reliability and consistency of results.

## 3. Mathematical modeling

The experimental data obtained from the experiment on water-vapor adsorption were used to study the relationship between the moisture content of each sample plotted against time, which gave the EMC. Moisture content (*M*_c_) denotes how much water is present in a sample, and was calculated using the following formula:5
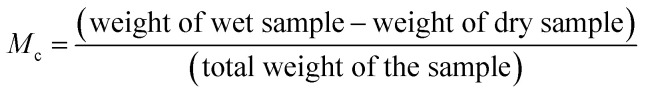


Moisture content is measured in (g g^−1^) or (mg g^−1^). The relationship between the EMC and water activity (*a*_w_) was also studied by using moisture-content data.

The adsorption-isotherm model gives information about the interaction between the adsorbate and adsorbent surface, the adsorption mechanism, the adsorbent capacity, and the performance of the overall adsorption process. We used three models (Langmuir, Freundlich, and Temkin) to evaluate adsorption performance. The Langmuir model is represented by the following equation:6
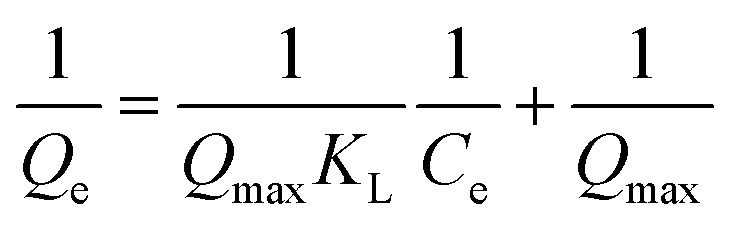
where *Q*_max_ is the adsorbent monolayer capacity (mg g^−1^), *Q*_e_ is the amount of absorbed adsorbate molecule per gram of absorbent (mg g^−1^), *C*_e_ is the adsorbate equilibrium concentration (mg g^−1^), and *K*_L_ is the Langmuir adsorption constant.

The Freundlich model is represented by the following equation:7
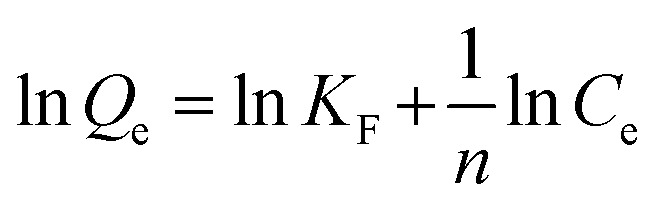
where *K*_F_ is the Freundlich constant, and *n* gives the value of the degree of linearity between the adsorbate and adsorption process.

The Temkin model is represented by the following equation:

8 *Q*_e_ = *B*_T_ ln *A*_T_ + *B*_T_ ln *C*_e_

where *A*_T_ is the binding equilibrium constant and *B*_T_ is the adsorption heat constant.^27^

### 3.1 Kinetic studies

The kinetics curves of synthesized materials for the adsorption of water vapors were evaluated by Lagergren's first-order adsorption kinetics model and second-order adsorption kinetics model. Both models were applied using certain parameters. The moisture content at time *t* and EMC can be calculated as:9
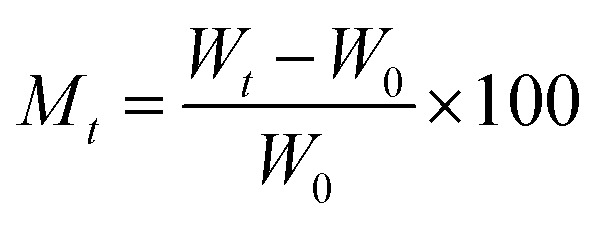
10
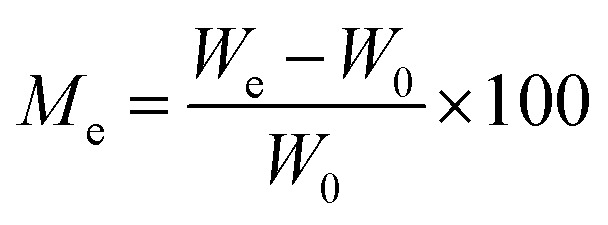
where *W*_0_ is the initial weight of the sample measured in grams, *W*_*t*_ is the weight of the sample at time *t*, and weight at equilibrium is denoted as *W*_e_.

The Lagergren first-order model and second-order adsorption kinetics model were applied to as-synthesized samples. The Lagergren second-order adsorption kinetics model fitted well on as-synthesized samples. The Lagergren second-order equation is described as:11
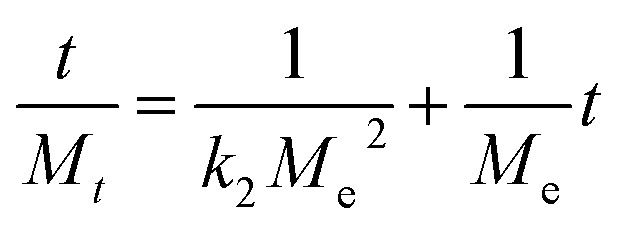
where *M*_*t*_ is the adsorption capacity at time *t*, *M*_e_ is the adsorption at equilibrium, *M*_0_ is the adsorption at *t* = 0, and *k*_2_ (min^−1^) is the adsorption rate constant for second-order kinetics.

### 3.2 Thermodynamic studies

Any thermodynamics study is based on several thermodynamic parameters: isosteric heat of adsorption, entropy, and Gibbs free energy. These parameters were calculated using the following equations.

12 *Q*_st_ = *q*_st_ + *H*_L_

13
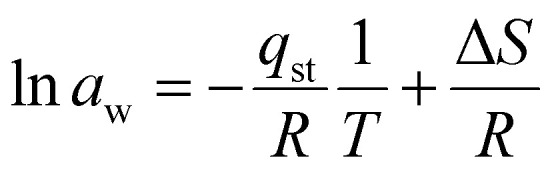

14 Δ*G* = *RT* ln *a*_w_

They give useful information about whether an adsorption process is exothermic, spontaneous, or favorable. The negative value of Gibbs free energy reveals that the adsorption process is spontaneous, and a positive value shows that the process is endothermic.^28^

## 4. Results and discussion

### 4.1 Characterization

The phase composition and crystallinity of synthesized materials were evaluated by XRD analysis. The obtained pattern and corresponding *hkl* values matched well with the standard pattern of zinc ferrite spinel (ICSD reference code number: 01-079-1150). The sharp and intense peaks had *hkl* values of (220), (311), (222), (511), and (440). The peaks having *hkl* values of (220) and (311) confirmed that the desired single-phase zinc ferrite and cobalt-doped zinc ferrite were obtained with the *Fd3̄m* space group.

Several parameters, such as the lattice parameter (*a*), cell volume (*V*_cell_), crystallite size (*D*_s_), and bulk density (*D*_x_), were obtained using XRD data. The calculated values of the lattice parameter, cell volume, and X-ray density of ZnFe_2_O_4_ were 8.47 Å, 608 Å^3^, and 5.27 g cm^−3^, whereas those of Zn_0.4_Co_0.6_Fe_2_O_4_ were 8.38 Å, 589 Å^3^, and 5.30 g cm^−3^, respectively. There is a decreasing trend for lattice parameter and cell volume with an increase in the cobalt content in ZnFe_2_O_4_ due to a decrease in the ionic size of Co^2+^ (0.72 Å) as compared with Zn^2+^ (0.82 Å) in a crystal lattice.^29^

**Fig. 2:**
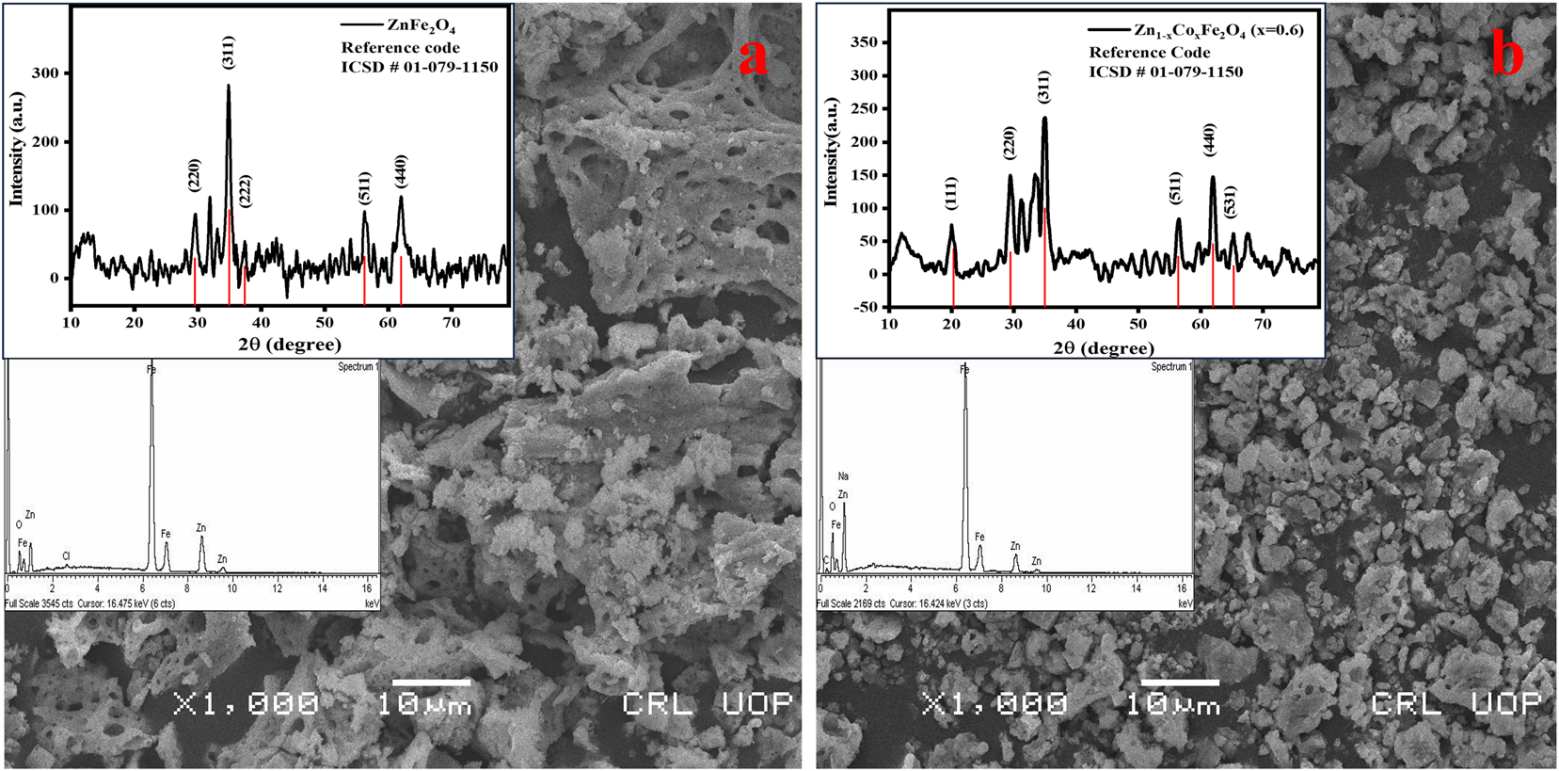
Scanning electron micrographs along with X-ray diffraction patterns and energy dispersive X-ray spectra for (a) ZnFe_2_O_4_ and (b) Zn_0.4_Co_0.6_Fe_2_O_4_.

The crystallite size of ZnFe_2_O_4_ and Zn_0.4_Co_0.6_Fe_2_O_4_ was observed using Scherrer's formula: it was 14 and 27 nm, respectively. The crystallite size of Zn_0.4_Co_0.6_Fe_2_O_4_ was greater than that of ZnFe_2_O_4_ because the increasing cobalt content in ZnFe_2_O_4_ particles leads to agglomeration. This phenomenon can be explained based the on-site preference of Zn^2+^, Co^2+^, and Fe^3+^ in a cubic unit cell. Zn^2+^ and Fe^3+^ have a strong preference towards tetrahedral and octahedral sites. Co^2+^ has a very strong preference towards tetrahedral and octahedral sites, and is uniformly distributed in the spinel structure. An increase in cobalt content forces Fe^3+^ towards tetrahedral sites, but this leads to an increase in the crystallite size of Zn_0.4_Co_0.6_Fe_2_O_4_ (28 nm) as compared with that of ZnFe_2_O_4_ (14 nm).^30^ The structure of ZnFe_2_O_4_ is a normal cubic structure because divalent Zn^2+^ occupies tetrahedral sites and trivalent Fe^3+^ occupies octahedral sites. Zn_0.4_Co_0.6_Fe_2_O_4_ shows a mixed structure due to the occupancy of cobalt ions at tetrahedral and octahedral sites. According to XRD data, an increase in the intensity of peaks at the (220) plane rather than the (440) plane was observed (Fig. 2). This phenomenon was due to the higher amount of cobalt ions present in tetrahedral rather than octahedral sites.^30,31^

SEM gives information about the surface morphology and effect of different compositions of dopants on the morphological properties of a material. The images obtained from SEM showed that spherical and uniform-sized particles had been formed. The obtained zinc ferrite nanoparticles showed agglomeration due to the magnetic interactions between different crystallites present in both samples. This occurred because the synthesized particles had higher surface energy, so particles combined and lowered their energy to attain the minimum surface area.^32^ Heating during the synthetic process can also affect crystallite agglomeration.^33^ In the case of Zn_0.4_Co_0.6_Fe_2_O_4_, the particle size increased and it underwent agglomeration of crystallites. The increase in crystallite size was also confirmed using XRD analysis.

Energy dispersive X-ray (EDX) spectroscopy was used to analyze the elemental composition of zinc ferrite spinels. The characteristic spectrum of zinc ferrite spinels confirmed the presence of Zn, Fe, and O elements and their respective atomic percentages. Spectra revealed that unwanted precursors such as nitrates and hydroxides were eliminated from the final product.^34^

**Fig. 3:**
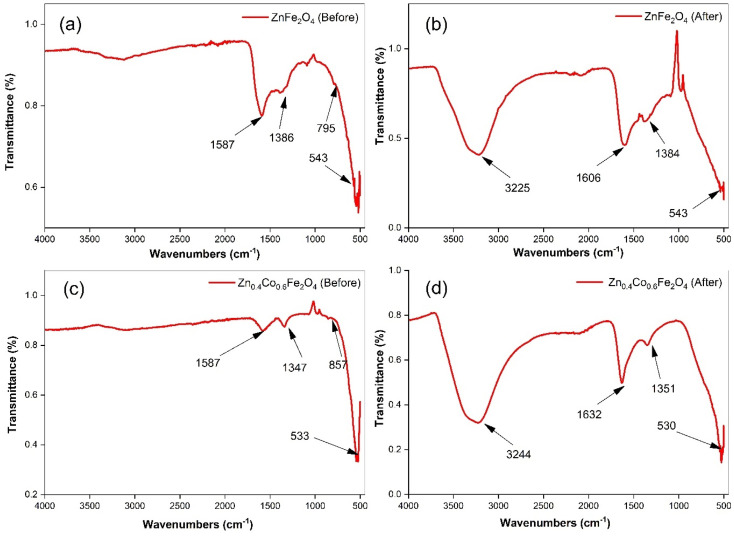
Fourier-transform infrared spectroscopy for ZnFe_2_O_4_ ((a) before and (b) after adsorption) and Zn_0.4_Co_0.6_Fe_2_O_4_ ((c) before & (d) after adsorption).

Fourier-transform infrared (FTIR) spectroscopy provided information about the formation of zinc ferrite and adsorption of water on the material surface. Initially, powder samples were washed with ethanol and DI water to remove impurities from the sample surface. Subsequently, experiments on water-vapor adsorption were conducted on ZnFe_2_O_4_ and Zn_0.4_Co_0.6_Fe_2_O_4_. FTIR spectroscopy of the samples before and after adsorption experiments are shown in Fig. 3.

The absorption band observed at 500–600 cm^−1^ was attributed to M–O vibrations at tetrahedral sites. A band observed below 500 cm^−1^ is associated with the vibration of M–O at octahedral sites. However, this band was not observed in our samples because our samples had bands at 500–4000 cm^−1^. Collectively, these two vibration bands confirmed the synthesis of zinc ferrite.^35^ The band attributed to M–O at the tetrahedral site shifted towards a lower wavenumber in the case of Zn_0.4_Co_0.6_Fe_2_O_4_. The band shifted with the variation in the cobalt-ion concentration; this may have been due to the redistribution of Zn, Co, and Fe onto tetrahedral and octahedral sites.^36,37^ The absorption bands for the bending and stretching vibrations of –OH owing to adsorbed moisture after harvesting experiments on zinc ferrite were observed at 1550–1650 and 3200–3300 cm^−1^, respectively.^38–41^ The bands at 1350–1386 cm^−1^ implied the existence of many residual hydroxyl groups.^42^ A clear indication of moisture adsorption could be confirmed by comparing FTIR spectra before and after harvesting experiments.

The magnetic properties of ZnFe_2_O_4_ and Zn_0.4_Co_0.6_Fe_2_O_4_ were evaluated by a vibrating sample magnetometer (VSM) with an applied magnetic field of 7000 Oe. Fig. S1† shows the hysteresis loop of prepared samples. Various magnetic parameters, such as saturation magnetization (*M*_s_), retentivity (*M*_r_), and magnetic moment (*μ*_B_), were calculated using a hysteresis loop (Table S1†). An important observation with reference to moisture harvesting was that changes in magnetic properties were not observed before and after harvesting experiments. Hence, the correlation of magnetic properties with the adsorbed moisture content was found to be least important. However, the magnetic properties of spinel ferrites are strongly influenced by cation distribution, particle size, and doping.^36^ With respect to the effects of dopants on the magnetic parameters of synthesized samples, ZnFe_2_O_4_ showed antiferromagnetic behavior and exhibited a saturation magnetization of 13.50 (emu g^−1^) before and after the adsorption of water molecules on the surface. This phenomenon was due to the nonmagnetic nature of Zn^2+^ (*μ*_B_ = 0),^43^ while ZnFe_2_O_4_ was weakly magnetic due to Fe^3+^ (*μ*_B_ = 5.9) residing at the octahedral site in the spinel structure.^44^

Compared with ZnFe_2_O_4_, Zn_0.4_Co_0.6_Fe_2_O_4_ showed ferromagnetic behavior with a saturation magnetization of 45.54 (emu g^−1^) due to the magnetic nature of Co^2+^ (*μ*_B_ = 3.87)^45^ and had a strong preference for tetrahedral and octahedral sites.^30^ This phenomenon leads to an increase in the non-equilibrium concentration of Fe^3+^ at tetrahedral and octahedral sites. Thus, oxygen vacancies are created by redistribution of Co^2+^ and Fe^3+^ at tetrahedral and octahedral sites. With the help of oxygen vacancies, some antiferromagnetic couplings (Fe_B_^3+^–O^2−^–Fe_B_^3+^) are converted to ferromagnetic couplings (Fe_B_–Fe_B_), so saturation magnetization increases.^46,47^ In the case of Zn_0.4_Co_0.6_·Fe_2_O_4_ (after adsorption), the overall saturation magnetization decreased due to a reduction in the number of oxygen vacancies as water molecules were adsorbed on these oxygen vacancies. The saturation magnetization in Zn_0.4_Co_0.6_·Fe_2_O_4_ (after adsorption) was due only to the redistribution of Co^2+^ and Fe^3+^.^45^

PZC gives information about surface charge. If the surface of a material becomes positively charged, it means that the negative part of water is attached to the surface. If the surface becomes negatively charged, it means that the positive part of the water is attached to the surface of the synthesized material. The same procedure as mentioned above was carried out for ZnFe_2_O_4_ and Zn_0.4_Co_0.6_Fe_2_O_4_ to obtain different PZC graphs (ESI Fig. 2). The presence of a positive charge on the surface of ZnFe_2_O_4_ and a negative charge on the surface of Zn_0.4_Co_0.6_Fe_2_O_4_ were observed (ESI Fig. 2†).

### 4.2 Adsorption studies

By applying the above-mentioned adsorption procedure, the data for the moisture content of synthesized ZnFe_2_O_4_ and Zn_0.4_Co_0.6_Fe_2_O_4_ were obtained. A graph of moisture content *versus* time was plotted using Excel™ (Microsoft).

**Fig. 4:**
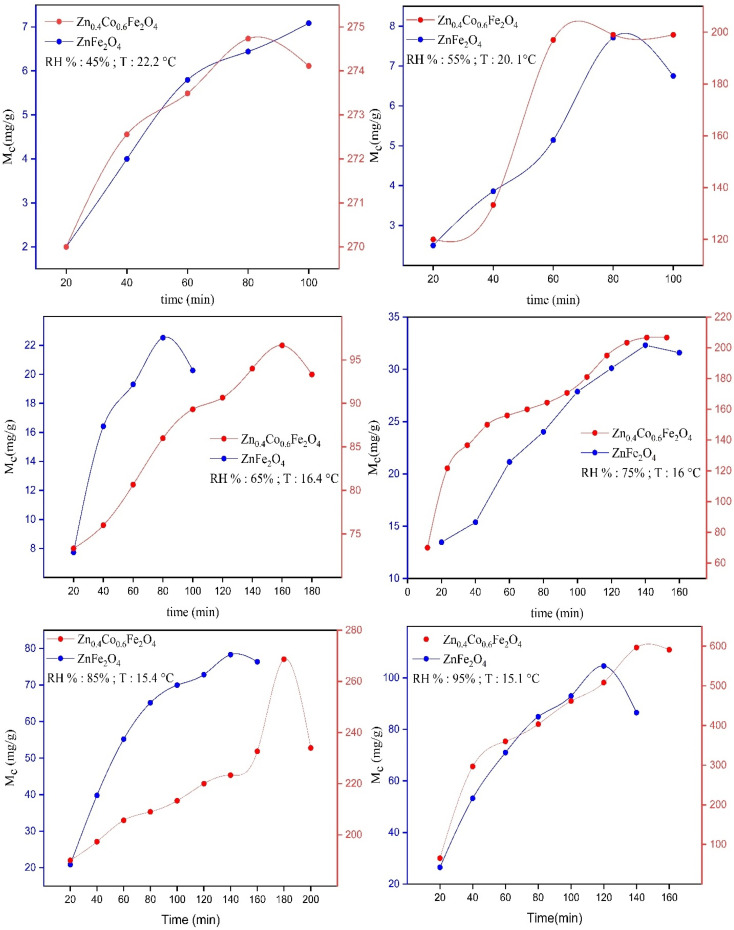
Graphs of moisture content (*M*_c_) *versus* time for synthesized ZnFe_2_O_4_ and Zn_0.4_Co_0.6_Fe_2_O_4_ at different RH percentages.

With respect to adsorption experiments, initially many water adsorption sites are vacant. After some time, these vacant sites are filled by water vapors and suddenly adsorption–desorption equilibrium is achieved. The EMC is defined as a state in which a material neither gains nor loses moisture. The point where adsorption–desorption equilibrium is achieved is known as the saturation point (*S*_p_). Beyond this point, water-vapor adsorption starts to decrease. Water-vapor adsorption varies according to humidity. If humidity increases, moisture content also increases due to the availability of more vapor towards the material surface at high humidity. At low humidity, the EMC and saturation point are achieved earlier due to less pressure applied by water vapors on the material surface. At high humidity, the pressure applied by water vapors on the material surface starts to increase; due to an increase in vapor pressure, more water vapors get adsorbed on the material surface so that the EMC and saturation point are achieved later. According to graphs, after attaining adsorption–desorption equilibrium, the values of moisture content started to decrease because the material had attained its saturation point, and its empty sites were filled (Fig. 4).

The maximum *M*_c_ observed for ZnFe_2_O_4_ was 104 ± 1 mg at 95% RH. The maximum *M*_c_ observed for Zn_0.4_Co_0.6_Fe_2_O_4_ was 596 ± 1 mg at 95% RH. These data suggested that different moisture content was obtained by ZnFe_2_O_4_ and Zn_0.4_Co_0.6_Fe_2_O_4_ at different RH percentages. The moisture content of Zn_0.4_Co_0.6_Fe_2_O_4_ increased as compared with that of ZnFe_2_O_4_ at different RH percentages because the redistribution of Fe^3+^ and Co^2+^ at octahedral sites led to an excess of charge being produced on the material surface. Hence, Zn_0.4_Co_0.6_Fe_2_O_4_ had a greater affinity for water-vapor adsorption as compared with that of ZnFe_2_O_4_.

The saturation point (*S*_p_) was also achieved very late in the case of Zn_0.4_Co_0.6_Fe_2_O_4_, but the redistribution of cobalt ions imparted an excess of charge on Zn_0.4_Co_0.6_Fe_2_O_4_. Hence, it took longer to adsorb all water molecules in the surroundings as compared with ZnFe_2_O_4_. A comparison between the saturation point and moisture content for ZnFe_2_O_4_ and Zn_0.4_Co_0.6_Fe_2_O_4_ at different RH percentages is given in Table 1.

**Table 1:** Comparison between the saturation point (*S*_p_, min) and moisture content (*M*_c_, mg g^−1^) for ZnFe_2_O_4_ and Zn_0.4_Co_0.6_Fe_2_O_4_ at different RH percentages

Relative humidity	45 ± 3%		55 ± 3%		65 ± 3%		75 ± 3%		85 ± 3%		95 ± 3%	
Zn_1−*x*_Co_*x*_Fe_2_O_4_	*S*_p_	*M*_c_	*S*_p_	*M*_c_	*S*_p_	*M*_c_	*S*_p_	*M*_c_	*S*_p_	*M*_c_	*S*_p_	*M*_c_
(*x* = 0.0)	80	7	80	7.7	80	23	140	32	140	78	120	104
(*x* = 0.6)	80	275	80	197	80	96	240	206	180	268	140	597

#### 4.2.1 Optimum conditions

A maximum water-adsorption capacity of 104 mg g^−1^ and 597 mg g^−1^ was achieved by ZnFe_2_O_4_ and Zn_0.4_Co_0.6_Fe_2_O_4_, respectively, at 95 ± 3% RH and 15 ± 2 °C. Overall gravimetric analysis (±1 mg) was used to determine the mass change before and after the adsorption of water vapors. The saturation point of Zn_0.4_Co_0.6_Fe_2_O_4_ was observed after placing a sample in an AWH plant for 140 min.

#### 4.2.2 Mechanism of action

Cobalt-doped ZnFe_2_O_4_ is considered to be a cost-effective material. If Co^2+^ is doped in ZnFe_2_O_4_, then Co^2+^ replaces Fe^3+^ that is already present in the octahedral site. Fe^3+^ moves towards tetrahedral sites if Zn^2+^ is present. The replacement between Fe^3+^ and Zn^2+^ creates some oxygen vacancies. Co^2+^ was uniformly distributed in tetrahedral and octahedral sites according to XRD analysis and FTIR spectroscopy. The release of oxygen molecules from the lattice maintained electroneutrality and created some oxygen vacancies according to VSM. Water molecules approach the material surface, fill the oxygen vacancies, deprotonation occurs, and hydroxyl ions are adsorbed onto the spinel surface. Hence, favorable adsorption occurs due to the overlapping of the e_g_ orbital of Co^2+^ and 2p orbital of the hydroxyl ion. Hydroxyl ions are responsible for further adsorption of water molecules.^48,49^ A possible bonding mechanism between water vapors at the surface is illustrated in the graphical abstract.

### 4.3 Equilibrium moisture content (*X*_eq_) *versus* water activity (*a*_w_)

**Fig. 5:**
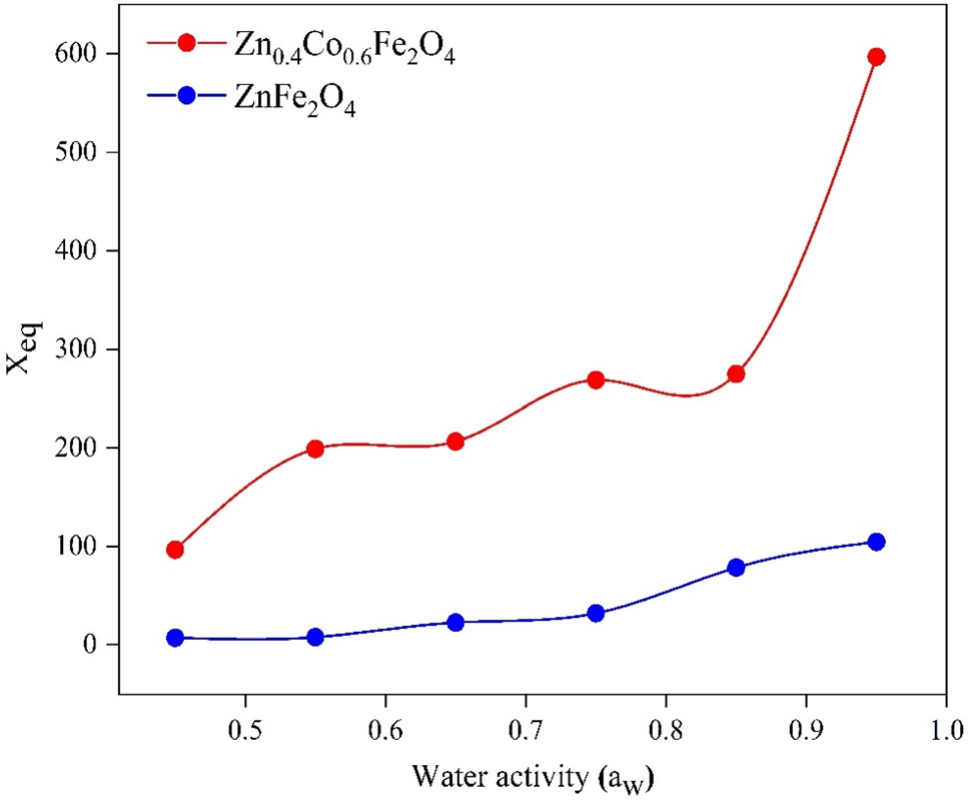
Equilibrium moisture content (*X*_eq_) *versus* water activity (*a*_w_) for ZnFe_2_O_4_ and Zn_0.4_Co_0.6_Fe_2_O_4_.

Using data for moisture content, the EMC was observed at the saturation point. We plotted a graph between the EMC and water activity (Fig. 5).

The EMC increased with increasing RH. *X*_eq_ increased initially at lower humidity (0.2–0.7) (Fig. 5). At this region, water molecules are strongly attracted to polar sites. In the region between 0.7 and 0.9, there was a steep trend between *X*_eq_ and *a*_w_ due to structural changes in the material. Beyond 0.9, *X*_eq_ became constant and no changes were observed in the material due to the occupation of all available sites by water molecules.^50^ The relationship between water activity and RH is described in eqn (15). The EMC for Zn_0.4_Co_0.6_Fe_2_O_4_ was higher as compared with that for ZnFe_2_O_4_ (Fig. 5) due to the strong affinity of Zn_0.4_Co_0.6_Fe_2_O_4_ for water molecules as compared with that of ZnFe_2_O_4_. Water activity is the ratio of water vapor pressure on a sample to the vapor pressure of pure water.15
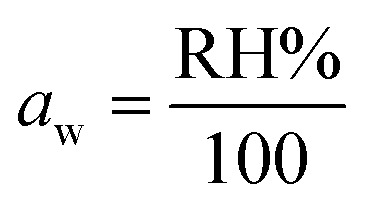


### 4.4 Adsorption isotherm models

**Fig. 6:**
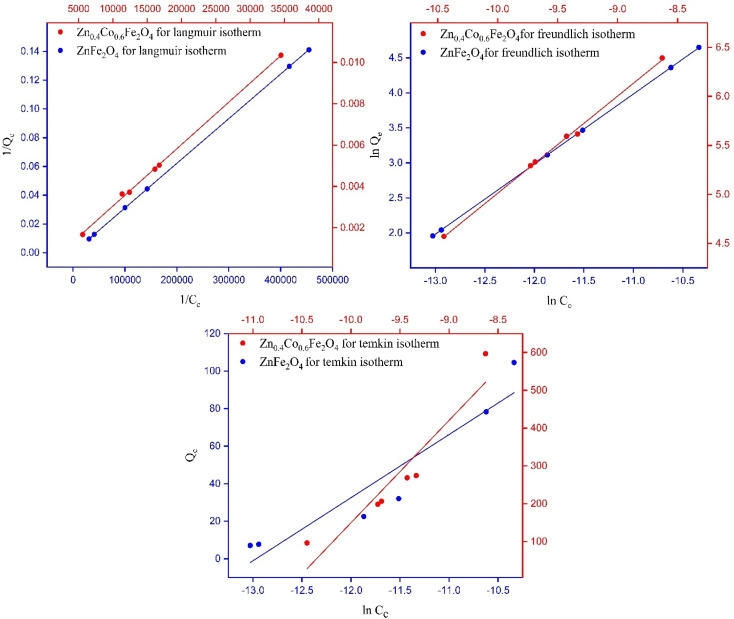
Fitting of experimental data to various isotherm models for ZnFe_2_O_4_ and Zn_0.4_Co_0.6_Fe_2_O_4_.

Every model has importance. For example, the Langmuir model and Freundlich model give information about linear adsorption or irreversible adsorption. The Temkin model gives information about whether a material shows physisorption or chemisorption behavior (Fig. 6).^27^

#### 4.4.1 Langmuir model

According to the Langmuir isotherm, a monolayer of adsorbate on the surface of the adsorbent provides the maximum adsorption capacity. *R*_L_ is a separation factor. It gives information that a material shows an unfavorable adsorption process (*R*_L_ > 1), linear adsorption process (*R*_L_ = 1), or Irreversible adsorption process (*R*_L_ = 0). The *R*_L_ for the synthesized materials was 1. Hence, the synthesized materials showed linear adsorption.^27^ The parameters for the Langmuir isotherm for synthesized materials are listed in Table 2.

**Table 2:** Kinetic and isothermal modeling parameters for ZnFe_2_O_4_ and Zn_0.4_Co_0.6_Fe_2_O_4_

Zn_1−*x*_Co_*x*_Fe_2_O_4_	Langmuir parameters		Freundlich parameters		Temkin parameters		75% ± 3%	85% ± 3%	95% ± 3%
Parameters	*K*_L_	*R*_L_	*K*_F_	*n*	*A*_T_	*B*_T_	*k*_2_	*k*_2_	*k*_2_
*x* = 0.0	139.03	0.9982	40.72	1.000	35.23	33.64	0.00330	0.00100	0.00030
*x* = 0.6	265.66	0.9785	40.58	1.009	28.68	270.78	0.00080	0.00300	0.00010

#### 4.4.2 Freundlich model

The Freundlich isotherm gives information about physical and chemical adsorption in which the adsorption process occurs in the form of a multilayer or monolayer. The Freundlich isotherm also assumes that the adsorption sites are heterogeneous. Also, *n* denotes the degree of linearity between the adsorbate and adsorption process. The following information was obtained by applying the Freundlich isotherm model.

• When *n* = 1, linear adsorption was shown.

• Adsorption is chemisorption if *n* < 1.

• Adsorption is physisorption if *n* > 1.

• Favorable adsorption can occur if 0 < 1/*n* < 1.

If *n* = 1 then all synthesized materials showed linear adsorption.^27^ The parameters of the Freundlich isotherm for synthesized materials are listed in Table 2.

#### 4.4.3 Temkin model

There are three basic assumptions for the Temkin model. As the surface area covered by the adsorbent increases, the heat of adsorption decreases linearly. There is a uniform distribution of binding energy on the surface of the absorbent, whereas the interaction involved in the adsorption process is between the adsorbate and absorbent. Here, *A*_T_ is the binding equilibrium constant, and *B*_T_ is the adsorption heat constant. *B*_T_ gives information about chemisorption or physisorption processes. Chemisorption occurs if *B*_T_ >8 kJ mol^−1^. Physisorption occurs if *B*_T_ <8 kJ mol^−1^. *B*_T_ >8 kJ mol^−1^ for as-synthesized materials, so both materials showed chemisorption behavior.^27^ The parameters for the Temkin isotherm for synthesized materials are listed in Table 2.

### 4.5 Kinetics studies

A plot of *t*/*M*_*t*_*versus* time (*t*) elicited a straight line and gave the values of intercept, slope, *M*_e_, and second-order rate constant. A higher value of the linear regression correlation coefficient (*R*^2^) was obtained using the second-order compared with the first-order kinetics model. Therefore, there was very good correlation between experimental data and parameters. The second-order kinetics model also provided information that the as-synthesized samples showed chemisorption behavior. The values for intercept, slope, experimental and theoretical *M*_e_, and second-order rate constant are given in Table 2.

**Fig. 7:**
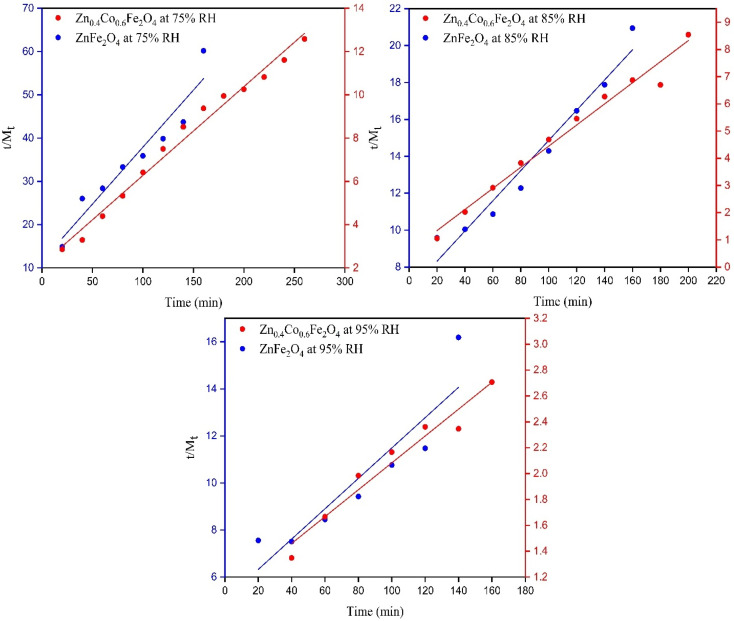
Fitting of experimental data to a pseudo second-order kinetic model for ZnFe_2_O_4_ and Zn_0.4_Co_0.6_Fe_2_O_4_ at 75%, 85%, and 95% RH.

There was a very small difference between the values of *M*_e_ calculated experimentally and theoretically, which showed the validity of the second-order kinetics model. There was a very low deviation at 55% RH. The maximum deviation was observed at 95% RH. Initially, the rate of vapor diffusion was high, and more sites were available so that the rate constant for the second-order reaction increased at 45% RH. After some time, most of the available sites became occupied by water molecules, and the diffusion of water vapors towards the material surface started to decrease so that the rate constant for second-order reaction started to decrease (Fig. 7).^51^

The rate constant (*k*_2_) decreased by increasing the humidity because equilibrium was achieved earlier. The fast adsorption rate was due to the attachment of water molecules with surface groups. At high humidity, the equilibrium was attained more slowly due to the lower availability of adsorption sites for water capture.^52^ The *k*_2_ for ZnFe_2_O_4_ and Zn_0.4_Co_0.6_Fe_2_O_4_ at 75%, 85%, and 95% RH are listed in Table 2.

### 4.6 Thermodynamic studies

#### 4.6.1 Isosteric heat of adsorption

The isosteric heat of sorption (*Q*_st_) is the energy released during the sorption of water molecules on the surface of an adsorbent. The isosteric heat of adsorption is equal to the addition of net isosteric heat of sorption (*q*_st_) and heat of vaporization of water (*H*_L_, 43 kJ mol^−1^).

16 *Q*_st_ = *q*_st_ + *H*_L_

It can be obtained from the Clausius–Clapeyron equation by plotting ln *a*_w_*versus* 1/*T*:17
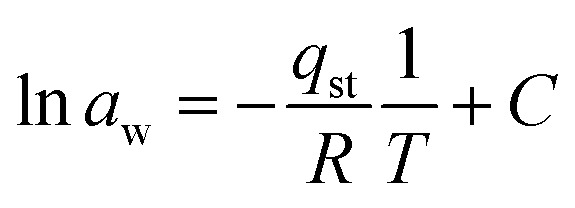
where *q*_st_ is the net isosteric heat of sorption (J mol^−1^), *T* is the absolute temperature, *R* is the general gas constant (8.314 J mol^−1^ K^−1^), and *C* is a constant.

The isosteric heat of adsorption was determined using the slope obtained from eqn (7).18
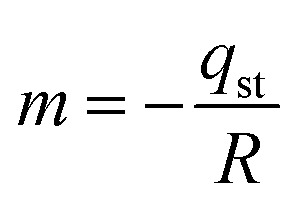
*m* = 7794.69; *R* = 8.314 J mol^−1^ K^−1^−*q*_st_ = 7794.69 × 8.314*q*_st_ = −64.805 kJ mol^−1^ put in **eqn (18)***Q*_st_ = −64.805 + 43*Q*_st_ = −21.805 kJ mol^−1^

#### 4.6.2 Sorption entropy

Entropy change gives information about energy analyses because it is proportional to the number of available sites for the adsorption process. Δ*S* can be calculated by plotting ln(*a*_w_) *versus* 1/*T* and the intercept is Δ*S*/*R* using eqn (19):19
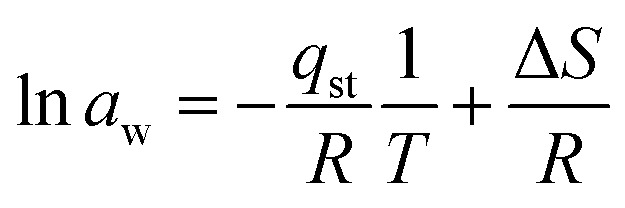
*c* = −27.20; *R* = 8.314 J mol^−1^ K^−1^20
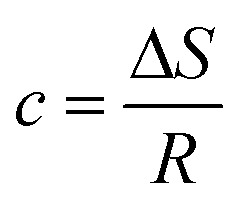
Δ*S* = −27.20 × 8.314Δ*S* = −0.2261 kJ mol^−1^

##### 4.6.2.1 Gibbs free energy

The Gibbs free energy gives the maximum amount of energy released in a process under constant pressure and temperature. The change in Gibbs free energy (Δ*G*) can help to determine if the process is spontaneous or not. Δ*G* at 16.4 °C and 75% RH was obtained using the following equation:

21 Δ*G* = *RT* ln *a*_w_

Δ*G* = 8.314 × 289.4 ln 0.75Δ*G* = −0.69 kJ mol^−1^

Δ*G* was negative so the adsorption process was spontaneous.

**Fig. 8:**
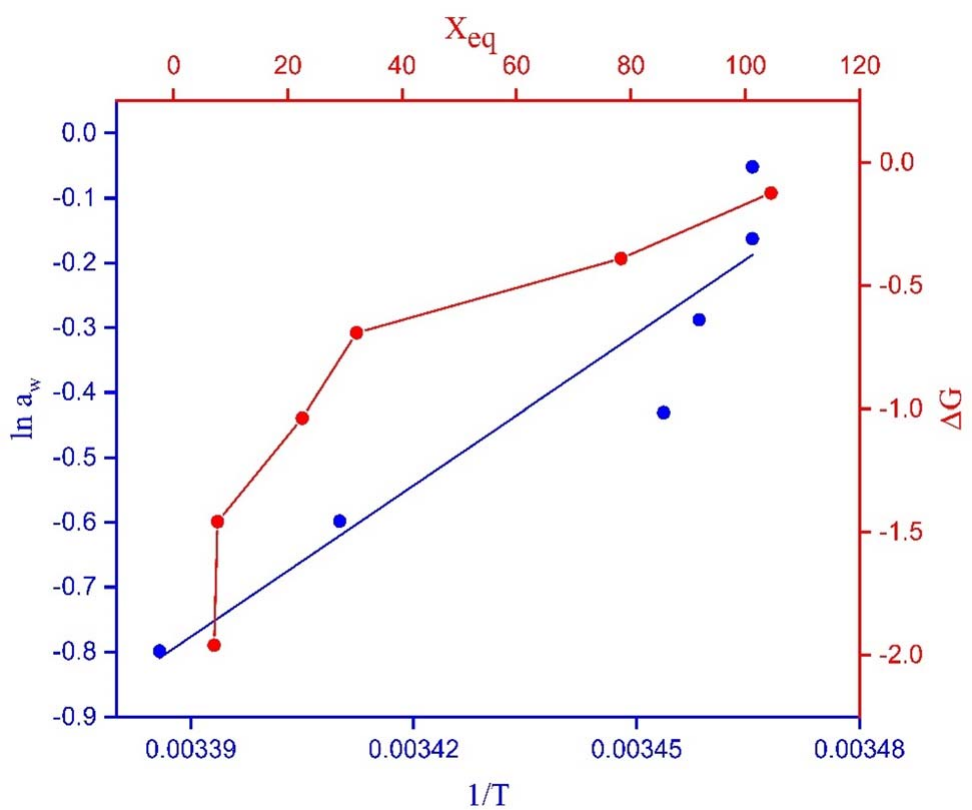
Relationship between the change in Gibbs free energy *versus X*_eq_ and ln *a*_w_*versus* 1/*T*.

The change in Gibbs free energy with the EMC is represented in Fig. 8. Δ*G* increased by increasing the EMC. Δ*G* varied from −1.95 to −0.12 kJ mol^−1^. The negative value confirmed that the process of water-vapor adsorption was spontaneous at different levels of humidity.^28^

#### 4.6.3 Elution and regeneration studies

Elution and regeneration of an adsorbent is an important factor that suggests the overall structural stability of a synthesized material after passing through a series of adsorption–desorption cycles.^16^ Elution studies were carried out using ZnFe_2_O_4_. Initially, 0.3 g of the sample was placed in an AWH plant at 95% RH. Adsorption experiments revealed ZnFe_2_O_4_ to show a moisture content of 104, 124, and 143 mg g^−1^ at three different cycles (ESI Fig. 3†). After that, desorption studies were carried out in a vacuum oven. The sample desorbed the whole moisture content rapidly at 45–60 °C.
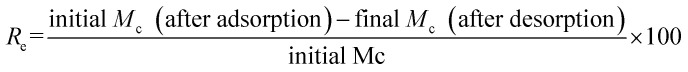


ZnFe_2_O_4_ showed high removal efficiency (94.7%). The overall removal efficiency was not 100% due to some water chemisorbed on the surface. Chemisorbed water was removed from the surface completely by placing the sample in an oven at 100 °C for 40 min. High removal efficiency shows high structural stability and reusability, and suggests an ideal material for AWH applications.^53–56^

## 5. Conclusions

Compared with conventional adsorbent materials, ZnFe_2_O_4_ and Zn_0.4_Co_0.6_Fe_2_O_4_ emerged as promising alternative materials for AWH due to cost-effectiveness, stability at a wide range of temperatures and humidity, environment friendliness, non-toxicity, and requirement for a lower regeneration temperature (up to 45–60 °C). ZnFe_2_O_4_ and Zn_0.4_Co_0.6_Fe_2_O_4_ were synthesized using a surfactant-assisted sol–gel method. Synthesized materials were confirmed by XRD analysis, EDX spectroscopy, and FTIR spectroscopy. The latter confirmed the adsorption of water vapors on the material surface. The EMC of ZnFe_2_O_4_ and Zn_0.4_Co_0.6_Fe_2_O_4_ were determined at different water activities (0.45–0.95). The EMC increased with increasing water activity. The maximum adsorption capacity for ZnFe_2_O_4_ and Zn_0.4_Co_0.6_Fe_2_O_4_ was 104 mg g^−1^ and 597 mg g^−1^ at 95% RH, respectively, in a closed AWH plant designed by our team. An adsorption capacity of 597 mg g^−1^ was achieved by Zn_0.4_Co_0.6_Fe_2_O_4_, which was even greater than the adsorption capacity of a zeolite (460 mg g^−1^) at 95% RH. The regeneration temperature for Zn_0.4_Co_0.6_Fe_2_O_4_ (45 °C) was much lower than that for the silica gel and a zeolite (100–200 °C). Results for isosteric heat of adsorption, Gibbs free energy, and sorption entropy suggested that the overall adsorption process was exothermic and spontaneous. Isotherm models (Langmuir and Freundlich) suggested that linear adsorption occurred between the adsorbate and adsorbent. The Temkin model suggested that the designed material showed chemisorption behavior. Kinetics studies suggested that the material underwent chemisorption behavior. Our results indicated that Zn_0.4_Co_0.6_Fe_2_O_4_ was an effective material for water capture compared with ZnFe_2_O_4_ in AWH applications.

## Conflicts of interest

There are no coflicts to declare.

## Supplementary Material

RA-014-D3RA08152D-s001

## References

[cit1] Badawi A. K., Bakhoum E. S., Zaher K. (2021). Arabian J. Sci. Eng..

[cit2] Srivastava S., Yadav A. (2018). Sol. Energy.

[cit3] Gleick P. H. , Water in crisis, Pacific Institute for Studies in Dev., Environment & Security, Stockholm Env. Institute, Oxford Univ. Press, 1993, p. 473

[cit4] Tan N. P. B., Ucab P. M. L., Dadol G. C., Jabile L. M., Talili I. N., Cabaraban M. T. I. (2022). Desalination.

[cit5] Waliullah R. M., Rehan A. I., Awual M. E., Rasee A. I., Sheikh M. C., Salman M. S., Hossain M. S., Hasan M. M., Kubra K. T., Hasan M. N., Marwani H. M., Islam A., Rahman M. M., Khaleque M. A., Awual M. R. (2023). J. Mol. Liq..

[cit6] Sheikh M. C., Hasan M. M., Hasan M. N., Salman M. S., Kubra K. T., Awual M. E., Waliullah R. M., Rasee A. I., Rehan A. I., Hossain M. S., Marwani H. M., Islam A., Khaleque M. A., Awual M. R. (2023). J. Mol. Liq..

[cit7] Awual M. E., Salman M. S., Hasan M. M., Hasan M. N., Kubra K. T., Sheikh M. C., Rasee A. I., Rehan A. I., Waliullah R. M., Hossain M. S., Marwani H. M., Asiri A. M., Rahman M. M., Islam A., Khaleque M. A., Awual M. R. (2024). J. Ind. Eng. Chem..

[cit8] Awual M. R., Hasan M. M., Shahat A., Naushad M., Shiwaku H., Yaita T. (2015). Chem. Eng. J..

[cit9] Batool A., Shah K. H., Hussain S., Hussain Z., Naqvi S. A. R., Sherazi T. A. (2022). Appl. Water Sci..

[cit10] Rasee A. I., Awual E., Rehan A. I., Hossain M. S., Waliullah R. M., Kubra K. T., Sheikh M. C., Salman M. S., Hasan M. N., Hasan M. M., Marwani H. M., Islam A., Khaleque M. A., Awual M. R. (2023). Surf. Interfaces.

[cit11] Awual M. R. (2015). Chem. Eng. J..

[cit12] Kubra K. T., Salman M. S., Hasan M. N., Islam A., Hasan M. M., Awual M. R. (2021). J. Mol. Liq..

[cit13] Awual M. R. (2017). Chem. Eng. J..

[cit14] Zhang S., Kuang J., Wei K., Zhou Y., Lei Y., Ma N., Miao H., Cao L. (2023). Colloids Surf., A.

[cit15] Awual M. R., Hasan M. N., Hasan M. M., Salman M. S., Sheikh M. C., Kubra K. T., Islam M. S., Marwani H. M., Islam A., Khaleque M. A., Waliullah R. M., Hossain M. S., Rasee A. I., Rehan A. I., Awual M. E. (2023). Sep. Purif. Technol..

[cit16] Awual M. E., Salman M. S., Hasan M. M., Hasan M. N., Kubra K. T., Sheikh M. C., Rasee A. I., Rehan A. I., Waliullah R., Hossain M. S. (2023). J. Ind. Eng. Chem..

[cit17] Rai P. K., Islam M., Gupta A. (2022). Sens. Actuators, A.

[cit18] Huang X., Qin Q., Ma Q., Wang B. (2022). Water.

[cit19] Bilal M., Sultan M., Morosuk T., Den W., Sajjad U., Aslam M. M. A., Shahzad M. W., Farooq M. (2022). Int. Commun. Heat Mass Transfer.

[cit20] He J., Yu H., Wang L., Yang J., Zhang Y., Huang W., Ouyang C. (2024). Eur. Polym. J..

[cit21] Chauhan P. R., Kaushik S. C., Tyagi S. K. (2022). Energy Convers. Manage.: X.

[cit22] Aghaie-Khafri M., Lafdani M. K. (2012). Powder Technol..

[cit23] Kumar V., Kumar N., Das S. B., Singh R. K., Sarkar K., Kumar M. (2021). Mater. Today: Proc..

[cit24] Vinosha P. A., Manikandan A., Ragu R., Dinesh A., Paulraj P., Slimani Y., Almessiere M. A., Baykal A., Madhavan J., Xavier B. (2021). Environ. Pollut..

[cit25] Vadivel M., Babu R. R., Ramamurthi K., Arivanandhan M. (2016). Ceram. Int..

[cit26] Gulicovski J. J., Čerović L. S., Milonjić S. K. (2008). Mater. Manuf. Processes.

[cit27] Ragadhita R., Nandiyanto A. B. D. (2021). Indones. J. Sci. Technol..

[cit28] Zhang Z.-S., Li X.-d., Jia H.-J., Liu Y.-l. (2022). Lwt.

[cit29] Ismail B., Hussain S. T., Akram S. (2013). Chem. Eng. J..

[cit30] Manikandan A., Kennedy L. J., Bououdina M., Vijaya J. J. (2014). J. Magn. Magn. Mater..

[cit31] Sathiyamurthy K., Rajeevgandhi C., Guganathan L., Bharanidharan S., Savithiri S. (2021). J. Mater. Sci.: Mater. Electron..

[cit32] Ansari A. A., Abushad M., Arshad M., Naseem S., Ahmed H., Husain S., Khan W. (2021). J. Mater. Sci.: Mater. Electron..

[cit33] Ansari M. R., Kem A., Agrohi P., Mallick P. K., Rao P., Peta K. R. (2023). Mater. Chem. Phys..

[cit34] Khan N.-u.-H., Gilani Z. A., Khalid M., Khan Asghar H. M. N. u. H., Hussain G., Shar M. A., Ali S. M., Khan M. A., Sheikh F. A., Alhazaa A. (2023). Phys. B.

[cit35] Sonia L. C., Phanjoubam S. (2023). Mater. Today: Proc..

[cit36] Andhare D. D., Patade S. R., Kounsalye J. S., Jadhav K. M. (2020). Phys. B.

[cit37] Patil K., Kadam S., Lokhande P., Balgude S., More P. (2021). Solid State Commun..

[cit38] Alfryyan N., Munir S., Latif M., Alrowaili Z. A., Al-Buriahi M. S., Irshad A., Suleman M. (2023). Optik.

[cit39] Alshammari A. H., Alshammari K., Alshammari M., Taha T. A. M. (2024). Int. J. Hydrogen Energy.

[cit40] Islam S., Lutfor Rahman M., Rassel Moni M., Biswas B., Farid Ahmed M., Sharmin N. (2023). Arabian J. Chem..

[cit41] Joshi A., Srivastava R. C. (2023). Mater. Today: Proc..

[cit42] Wang M., Huang Y., Chen X., Wang K., Wu H., Zhang N., Fu H. (2017). J. Alloys Compd..

[cit43] Rosales-González O., Bolarín-Miró A. M., Cortés-Escobedo C. A., Pedro-García F., Patiño-Pineda J. A., Sánchez-De Jesús F. (2023). Ceram. Int..

[cit44] Sun Y. , Deng X., Zong Y., Li X., Zhang J., Feng J., Chi X., Shi Z., Zheng X. and Peng Y., 2021

[cit45] Tatarchuk T. R., Paliychuk N. D., Bououdina M., Al-Najar B., Pacia M., Macyk W., Shyichuk A. (2018). J. Alloys Compd..

[cit46] Zuo X., Zhang D., Zhang J., Fang T. (2023). Ceram. Int..

[cit47] Yadav B. S., Vishwakarma A. K., Singh A. K., Kumar N. (2023). Vacuum.

[cit48] Kim J., Yin X., Tsao K.-C., Fang S., Yang H. (2014). J. Am. Chem. Soc..

[cit49] Hasan M. N., Salman M. S., Hasan M. M., Kubra K. T., Sheikh M. C., Rehan A. I., Rasee A. I., Awual M. E., Waliullah R. M., Hossain M. S., Islam A., Khandaker S., Alsukaibi A. K. D., Alshammari H. M., Awual M. R. (2023). J. Mol. Struct..

[cit50] Sablani S. S., Bruno L., Kasapis S., Symaladevi R. M. (2009). J. Food Eng..

[cit51] Alkan M., Demirbaş Ö., Doğan M. (2007). Microporous Mesoporous Mater..

[cit52] Mittal H., Al Alili A., Alhassan S. M. (2021). J. Environ. Chem. Eng..

[cit53] Kubra K. T., Hasan M. M., Hasan M. N., Salman M. S., Khaleque M. A., Sheikh M. C., Rehan A. I., Rasee A. I., Waliullah R. M., Awual M. E., Hossain M. S., Alsukaibi A. K. D., Alshammari H. M., Awual M. R. (2023). Colloids Surf., A.

[cit54] Salman M. S., Sheikh M. C., Hasan M. M., Hasan M. N., Kubra K. T., Rehan A. I., Awual M. E., Rasee A. I., Waliullah R. M., Hossain M. S., Khaleque M. A., Alsukaibi A. K. D., Alshammari H. M., Awual M. R. (2023). Appl. Surf. Sci..

[cit55] Salman M. S., Hasan M. N., Hasan M. M., Kubra K. T., Sheikh M. C., Rehan A. I., Waliullah R. M., Rasee A. I., Awual M. E., Hossain M. S., Alsukaibi A. K. D., Alshammari H. M., Awual M. R. (2023). J. Mol. Struct..

[cit56] Kubra K. T., Salman M. S., Hasan M. N., Islam A., Teo S. H., Hasan M. M., Sheikh M. C., Awual M. R. (2021). J. Mol. Liq..

