# Supplementary material for: Exploring moisture adsorption on cobalt-doped ZnFe2O4 for applications in atmospheric water harvesting
Source: RSC Adv. 2024 Feb 19;14(9):6165–77. doi: 10.1039/d3ra08152d (PMC10875414; doi:10.1039/d3ra08152d)
Supplement: RA-014-D3RA08152D-s001 [file RA-014-D3RA08152D-s001.pdf]

Electronic Supplementary Material (ESI) for RSC Advances.
This journal is © The Royal Society of Chemistry 2024

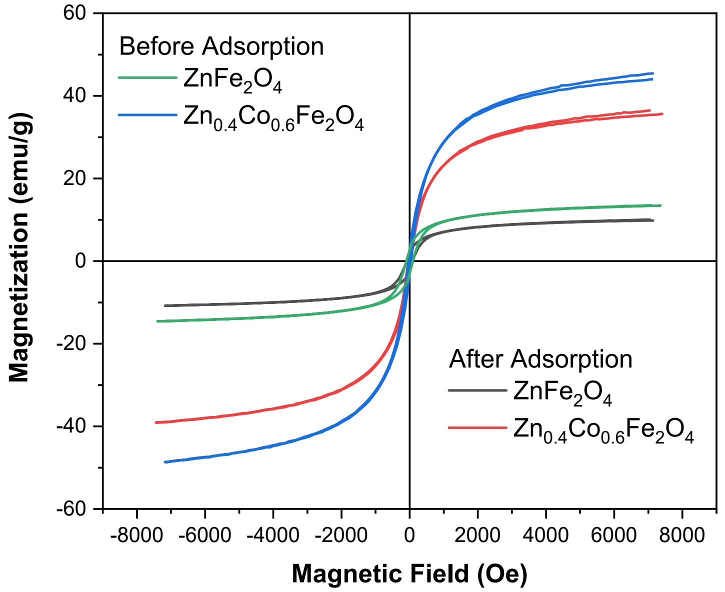

Supplementary figure 1: Magnetization hysteresis loop of $ZnFe_2O_4$ and $Zn_{1-x}Co_xFe_2O_4$ (x=0.6) before and after adsorption

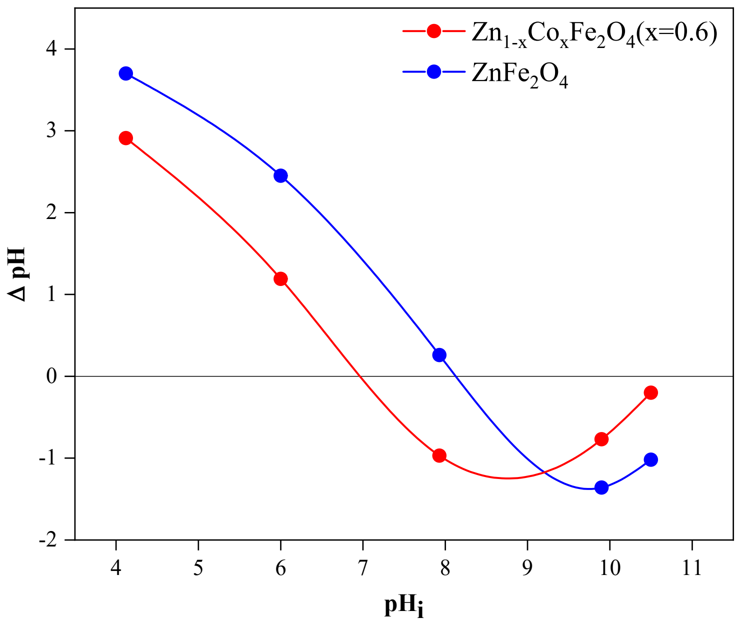

Supplementary figure 2: Change in pH versus initial pH for $ZnFe_2O_4$ and $Zn_{1-x}Co_xFe_2O_4$ (x=0.6)

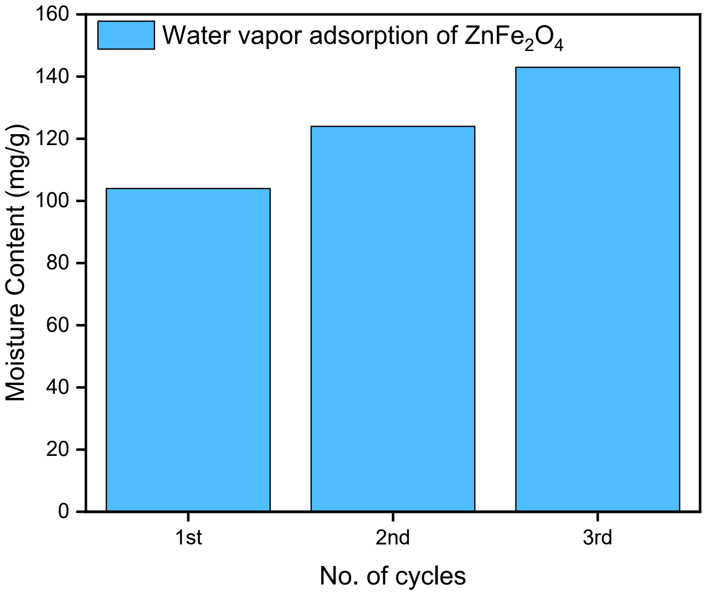

Supplementary figure 3: Regeneration studies of $ZnFe_2O_4$ at various cycles

Table S1: Magnetic parameters for $ZnFe_2O_4$ (Before and after adsorption) and $Zn_{0.4}Co_{0.6}Fe_2O_4$ (Before and after adsorption)

Sample	Saturation magnetization M_s (emu/g)	Retentivity M_r (emu/g)	Coercivity H_c (Oe)	Squareness ratio $R= M_r/M_s$	Magneton number n_B (μ_B)
**$ZnFe_2O_4$ (Before adsorption)**	13.50113	2.73646	94.71329	0.20268	0.58277
**$ZnFe_2O_4$ (After adsorption)**	13.59021	2.75441	93.38768	0.20268	0.586615
**$Zn_{0.4}Co_{0.6}$ Fe_2O_4 (Before adsorption)**	45.54298	2.55538	35.54602	0.05611	1.9343
**$Zn_{0.4}Co_{0.6}$ Fe_2O_4 (After adsorption)**	36.42958	2.09884	36.30427	0.05761	1.547236

$ZnFe_2O_4$:

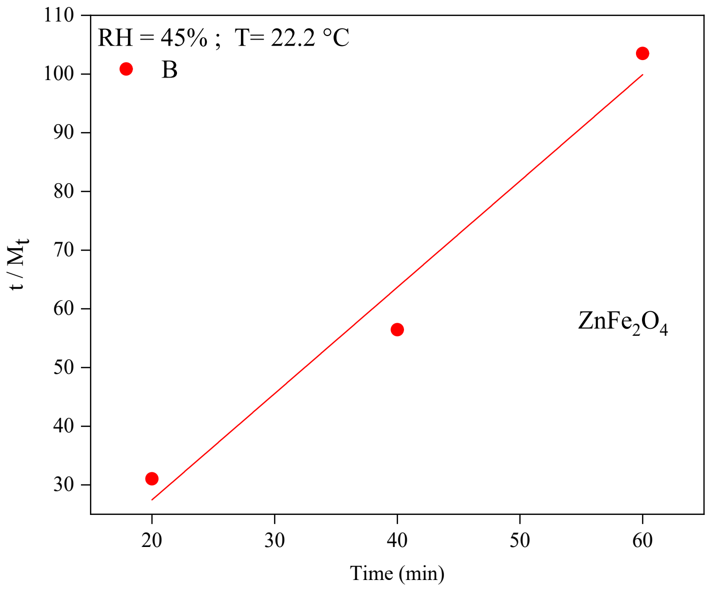

Fig: 4.38 Pseudo 2nd order kinetics model for synthesized $ZnFe_2O_4$ at a RH of 45 %

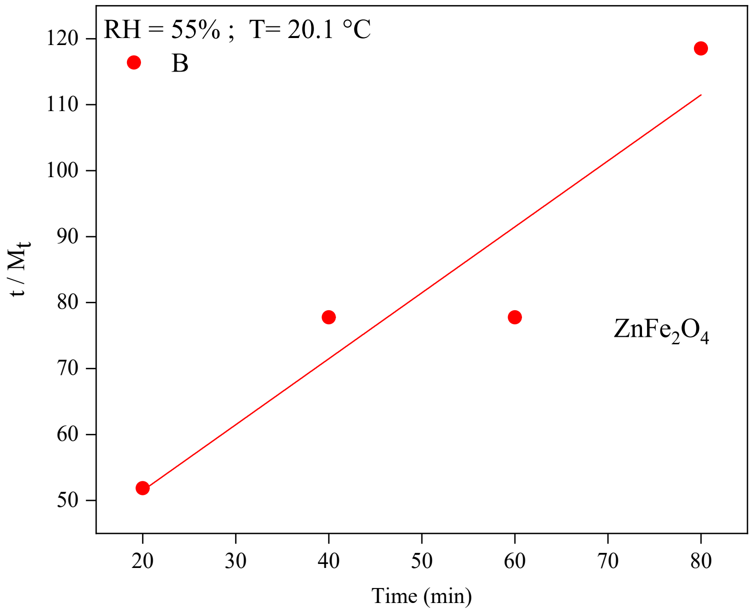

Fig: 4.39 Pseudo 2nd order kinetics model for synthesized $ZnFe_2O_4$ at a RH of 55 %

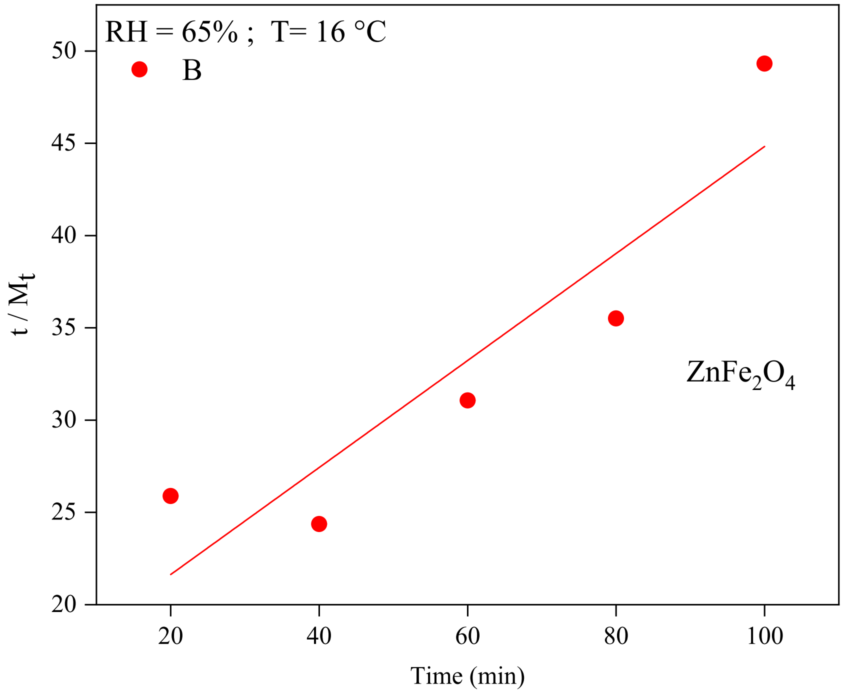

Fig: 4.40 Pseudo 2nd order kinetics model for synthesized ZnFe2O4 at a RH of 65 %

Parameters for Pseudo 2nd order kinetics model of synthesized $Zn_{1-x}Co_xFe_2O_4$ (x=0.6) at different RH %

Sr No	Humidity	Slope (m)	Intercept (c)	Me (Th)	Me (Exp) (mg/g)	k_2 (min^{-1})
1.	45 % ± 3%	1.8113	-8.7818	0.5521	0.7085	-0.3736
2.	55 % ± 3%	0.9999	31.4804	1.009	0.7714	0.0318
3.	65 % ± 3%	0.2899	15.8338	3.4495	2.2530	0.0053
4.	75 % ± 3%	0.2173	14.3300	4.6019	3.2031	0.0033
5.	85 % ± 3%	0.0818	6.6828	12.2249	7.8305	0.0010

6.	95 % ± 3%	0.0433	6.1666	23.0947	10.4550	0.0003

$Zn_{1-x}Co_xFe_2O_4$ (x=0.6):

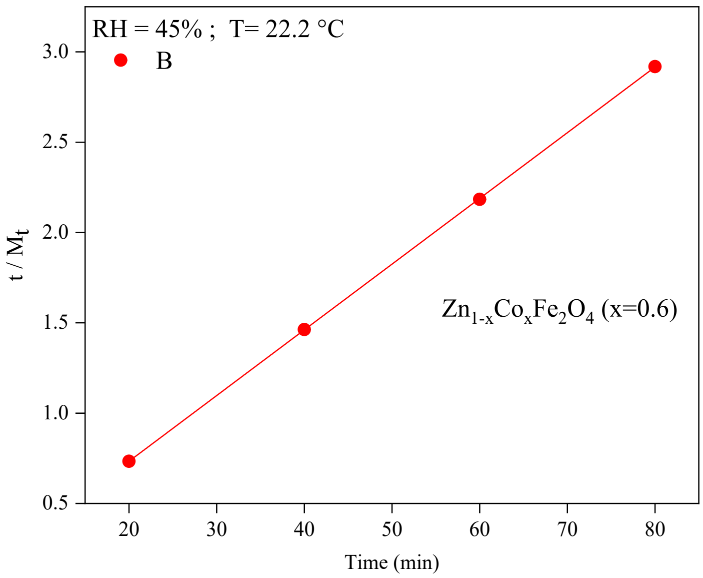

Fig:4.56 Pseudo 2nd order kinetics model for synthesized $Zn_{1-x}Co_xFe_2O_4$ (x=0.6) at a RH of 45 %

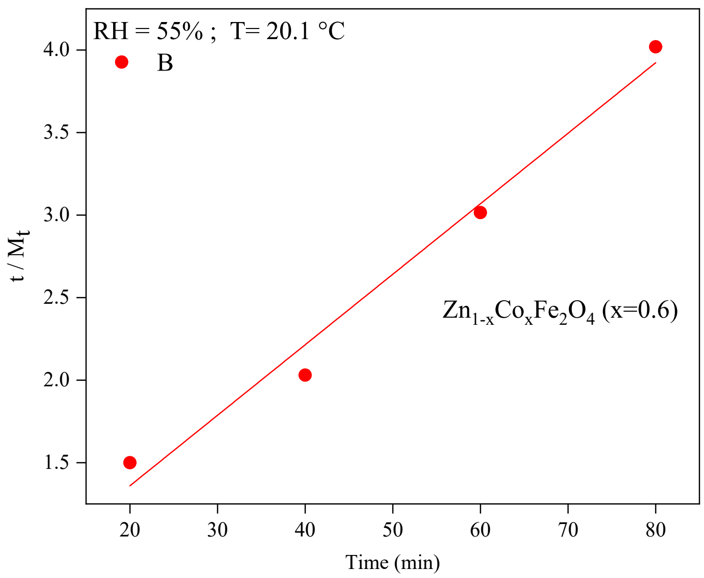

Fig:4.57 Pseudo 2nd order kinetics model for synthesized $Zn_{1-x}Co_xFe_2O_4$ (x=0.6) at a RH of 55 %

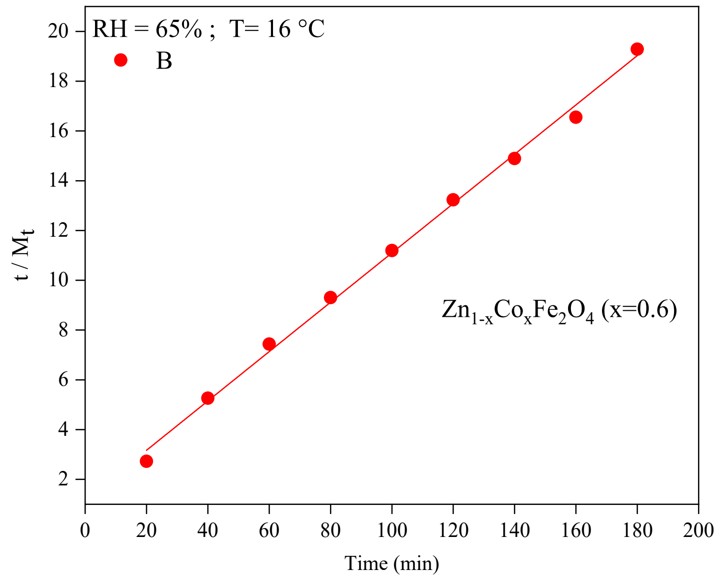

Fig:4.58 Pseudo 2nd order kinetics model for synthesized $Zn_{1-x}Co_xFe_2O_4$ (x=0.6) at a RH of 65 %

Parameters for Pseudo 2nd order kinetics model of synthesized $Zn_{1-x}Co_xFe_2O_4$ (x=0.6) at different RH %

Sr No	Humidity	Slope (m)	Intercept (c)	Me (Th)	Me(exp) (mg/g)	k_2 (min^{-1})
1.	45 % ± 3 %	0.0364	0.0058	27.4725	27.4736	0.2285
2.	55 % ± 3 %	0.0427	0.5052	23.4192	19.9001	0.0036
3.	65 % ± 3 %	0.0991	1.1871	10.0910	9.6667	0.0083
4.	75 % ± 3 %	0.0410	2.1683	24.3902	20.6667	0.0008
5.	85 % ± 3 %	0.0388	0.5677	25.7732	26.8667	0.0030
6.	95 % ± 3 %	0.0104	1.0446	96.1538	59.6667	0.0001
